# 8,9-Epoxyeicosatrienoic Acid Inhibits Antibody Production of B Lymphocytes in Mice

**DOI:** 10.1371/journal.pone.0040258

**Published:** 2012-07-03

**Authors:** Yanxiang Gao, Juan Feng, Kongyang Ma, Zhou Zhou, Yi Zhu, Qingbo Xu, Xian Wang

**Affiliations:** 1 Department of Physiology and Pathophysiology, School of Basic Medical Sciences, Peking University, Key Laboratory of Molecular Cardiovascular Science, Ministry of Education, Beijing, People’s Republic of China; 2 Cardiovascular Division, Kings College London BHF Centre, London, United Kingdom; University of Southern California, United States of America

## Abstract

Epoxyeicosatrienoic acids (EETs), synthesized from arachidonic acid by cytochrome P450 epoxygenases, are converted to dihydroxyeicosatrienoic acids by soluble epoxide hydrolase. EETs exert anti-inflammatory effects. However, the effect of EETs on humoral immunity is poorly understood. The present study is to investigate the potential role of EETs on B cell function and mechanisms. We examined the role of EETs on antibody production of splenic B cells from C57BL/6 and apolipoprotein E-deficient (ApoE−/−) mice by means of ELISA. Of the 4 EET regioisomers, 8,9-EET decreased basal and activation-induced B cell antibody secretion. As well, 8,9-EET significantly inhibited B-cell proliferation and survival, plasma cell differentiation and class-switch recombination. Western blot analysis revealed that lipopolysaccharide-induced nuclear translocation of NF-κB could be attenuated by 8,9-EET. Furthermore, germinal center formation was impaired by 8,9-EET in mice *in vivo*. 8,9-EET may inhibit B-cell function *in vitro* and *in vivo,* which suggests a new therapeutic strategy for diseases with excess B cell activation.

## Introduction

B cells play an essential role in immunity [1]. The differentiation of B cells into antibody-secreting plasma cells is essential for humoral immune responses. After antigen-stimulation in secondary lymphoid organs, naive B cells proliferate and undergo maturation and differentiation that includes class switch recombination, affinity maturation, and differentiation into plasma cells or memory B cells [1], [2]. Effective antibody response depends on the integration of multiple signals. Although engagement of B cell receptor by specific antigens initiates the cascade, non-antigen–specific stimuli, such as lipopolysaccharide (LPS) and CD40L, have a profound effect on the quantity and quality of the response [3], [4]. Activation of mouse B cells by LPS induces B cell differentiation as well as increased antibody production [3]. In addition, the maturation and differentiation of B cell depend on concerted action of panoply of transcription factors, most notably interferon regulatory factor 4 (IRF-4), X-box binding protein 1 (XBP-1) and activation-induced cytidine deaminase (AICDA), which leads to the gene expressions necessary for plasma cell differentiation and class switch recombination of B cells [5]–[7].

Epoxyeicosatrienoic acids (EETs) are cytochrome P450 (CYP 450) metabolites synthesized from the essential fatty acid arachidonic acid. They include four regioisomers, 5,6-, 8,9-, 11,12-, and 14,15- EET, and are converted to dihydroxyeicosatrienoic acids by soluble epoxide hydrolase (sEH) [8]. Early studies indicate that EETs produce vascular relaxation [9]. Subsequent studies indicate that EETs exert other multiple beneficial biological functions, including angiogenesis, smooth muscle antimigratory, fibri-nolysis, hormone secretion, bronchodilation, anti-inflammatory and anti-atherosclerosis effects [10]–[17].

Arachidonic acid is abundant in immune cells and is composed of 15∼20% of fatty acid in phospholipids of the plasma membrane [18]. Many studies have showed that cyclooxygenase and lipoxygenase metabolites of arachidonic acid contribute to cell and humoral immunity [19]–[22], but the effect of the third major class of arachidonic acid metabolites is little known. CYP 450 epoxygenases and sEH are found in lymphoid tissues, such as spleen and lymph nodes [23], which suggest the biological roles of EETs in immunity. In addition, two studies have shown that CYP450 epoxygenase product 5,6-EET is responsible for hypotonicity-induced responses in B cells [24], [25]. However, the effect of EETs on humoral immunity is little known.

In the present study, we investigated the potential role of EETs in the function of splenic B cells from C57BL/6 and ApoE−/− mice. 8,9-EET inhibited B cell to proliferate, survival, plasmacytoid cell generation, class-switch recombination, and antibody secretion, which might be mediated by the inhibition of NF-κB activation. This knowledge might contribute to the treatment of diseases induced by overactivated B cells.

## Results

### 8,9-EET Decreased Antibody Production by B Cells in C57BL/6 and ApoE−/− Mice

**Figure 1:**
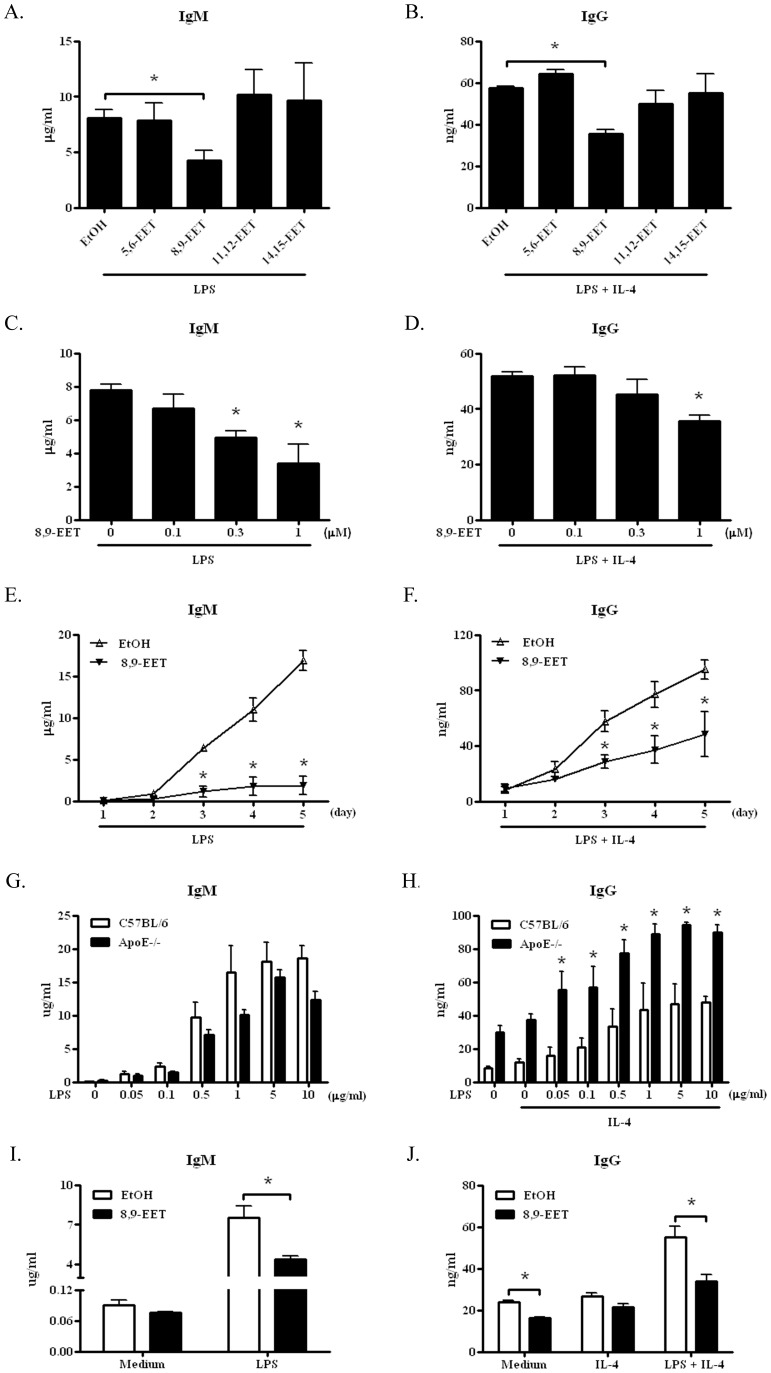
8,9-epoxyeicosatrienoic acid (EET) inhibited B-cell antibody production in C57BL/6 and ApoE−/− mice. ELISA of levels of IgM and IgG (A and B) in the supernatant of cultured B cells from C57BL/6 mice after incubation for 3 days with 1 µM EETs plus 5 µg/ml lipopolysaccharide (LPS) with or without 50 ng/ml IL-4. ELISA of levels of IgM (C and E) and IgG (D and F) in the supernatant of cultured B cells from C57BL/6 mice with indicated doses of 8,9-EET (C and D) or with 1 µM 8,9-EET for the indicated times (E and F) with 5 µg/ml LPS with or without 50 ng/ml IL-4. (G and H) ELISA of production of IgM and IgG by B cells from ApoE−/− mice and age- and sex-matched C57BL/6 control mice after incubation for 3 days with the indicated doses of LPS with or without IL-4 (50 ng/ml). *, *P*<0.05 vs. C57BL/6 control mice. (I and J) ELISA of IgM and IgG secretions by cultured B cells in ApoE−/− mice after incubation for 3 days with 1 µM 8,9-EET and/or 5 µg/ml LPS with or without IL-4 (50 ng/ml). Data are means ± SEM from 3 independent experiments. *, *P*<0.05 vs. time or day 0 or no 8,9-EET.

To investigate the potential effect of EETs on peripheral B cells, purified B cells were cultured with or without EETs, and IgM and IgG production was detected in the presence of 5 µg/ml LPS and/or 50 ng/ml IL-4. As compared with controls, 8,9-EET (1 µM) but not 5,6-, 11,12- or 14,15-EET significantly decreased the production of IgM (4.2±0.9 vs. 8.0±0.7 µg/ml) and IgG (35.6±1.8 vs. 57.4±1.0 ng/ml) (Figure 1A and 1B). Compared with different classical stimuli including anti-IgM F(ab)_2_ and sCD40L to activate B cells, the decreased IgM and IgG production by 8,9-EET was greater with LPS and LPS plus IL-4 stimulation (data not shown). In addition, antibody production was downregulated by 8,9-EET in a concentration- and time-dependent manner (Figure 1C–1F).

We examined the effect of 8,9-EET on B cell antibody production in ApoE−/− mice, the spontaneous model of atherosclerosis, with a genetic background of C57BL/6 mice. First, we compared the antibody levels of naïve and LPS-activated B cells from ApoE−/− mice and age- and sex-matched C57BL/6 mice. Secretion of IgG but not IgM by LPS-activated B cells was significantly higher, by more than 2-fold, in ApoE−/− mice than C57BL/6 control mice (Figure 1G and 1H). We then treated B cells of ApoE−/− mice with 1 µM 8,9-EET and/or 5 µg/ml LPS with or without 50 ng/ml IL-4 for 3 days. As compared with the control, 8,9-EET markedly decreased the production of IgM (4.4±0.2 vs. 7.5±0.5 µg/ml) and IgG (34.0±3.1 vs. 55.2±5.0 ng/ml) in LPS-activated B cells from ApoE−/− mice (Figure 1I and 1J), so 8,9-EET inhibited antibody production from normal B-cells and hyper-responsive one from ApoE−/− mice.

### 8,9-EET Inhibited B-cell Proliferation and Survival

**Figure 2:**
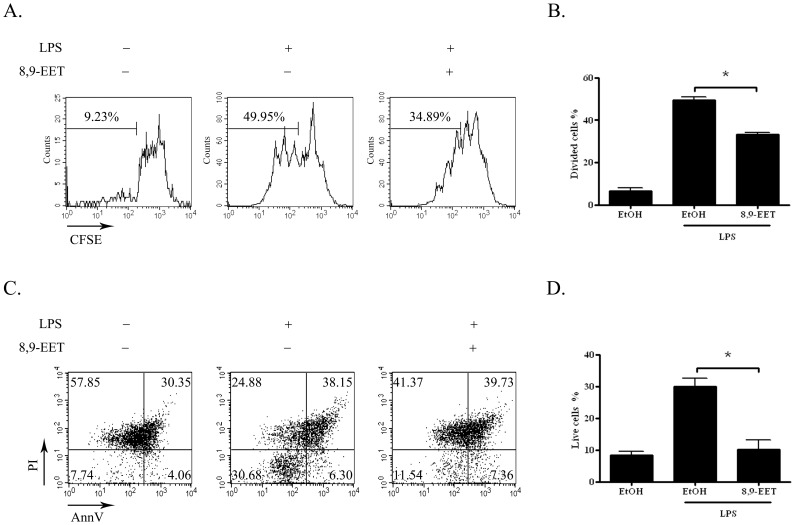
8,9-EET attenuated B-cell proliferation and survival. A. Splenic B cells were prelabeled with carboxyfluorescein succinimidyl ester (CFSE) and stimulated with 5 µg/ml LPS with or without 8,9-EET (1 µM) for 72 hr. Cell proliferation was monitored by measuring the dilution of CFSE. B. Quantification of 3 independent experiments of proliferation assay. C. Splenic B cells were stimulated with 5 µg/ml LPS with or without 8,9-EET (1 µM) for 48 hr. Flow cytometry of cell survival after annexin V (AnnV) and propidium iodide (PI) staining of cultured B cells. D. Quantification of three independent experiments of survival. Data are expressed as means ± SEM. *, *P*<0.05 vs. no 8,9-EET.

To investigate the mechanism of decreased antibody production with 8,9-EET, we monitored cell division by carboxyfluorescein succinimidyl ester (CFSE) dilution assay. CFSE-labeled B cells from C57BL/6 mice were cultured under various conditions for 3 days and then analyzed by flow cytometry. The proportion of dividing cells was lower with 8,9-EET (1 µM) plus LPS (5 µg/mL) than with LPS alone (33.2±0.9% vs. 49.3% ±1.7%) (Figure 2A and 2B). We next examined the effect of 8,9-EET on B-cell survival stimulated with LPS. B cells cultured for 2 days after stimulation were stained with annexin V (AnnV) and propidium iodide (PI) to identify live cells (AnnV and PI double-negative cells). The percentage of live cells was substantially lower with 8,9-EET plus LPS than with LPS alone (10.2% ±3.0% vs. 29.9% ±2.6%) (Figure 2C and 2D). Similarly, 8,9-EET inhibited the LPS-induced proliferation and survival of B cells from ApoE−/− mice (data not shown). Therefore, decreased B-cell proliferation and survival might contribute to the inhibition of antibody production by 8,9-EET.

### Plasma-cell Differentiation of B Cells was Inhibited by 8,9-EET

**Figure 3:**
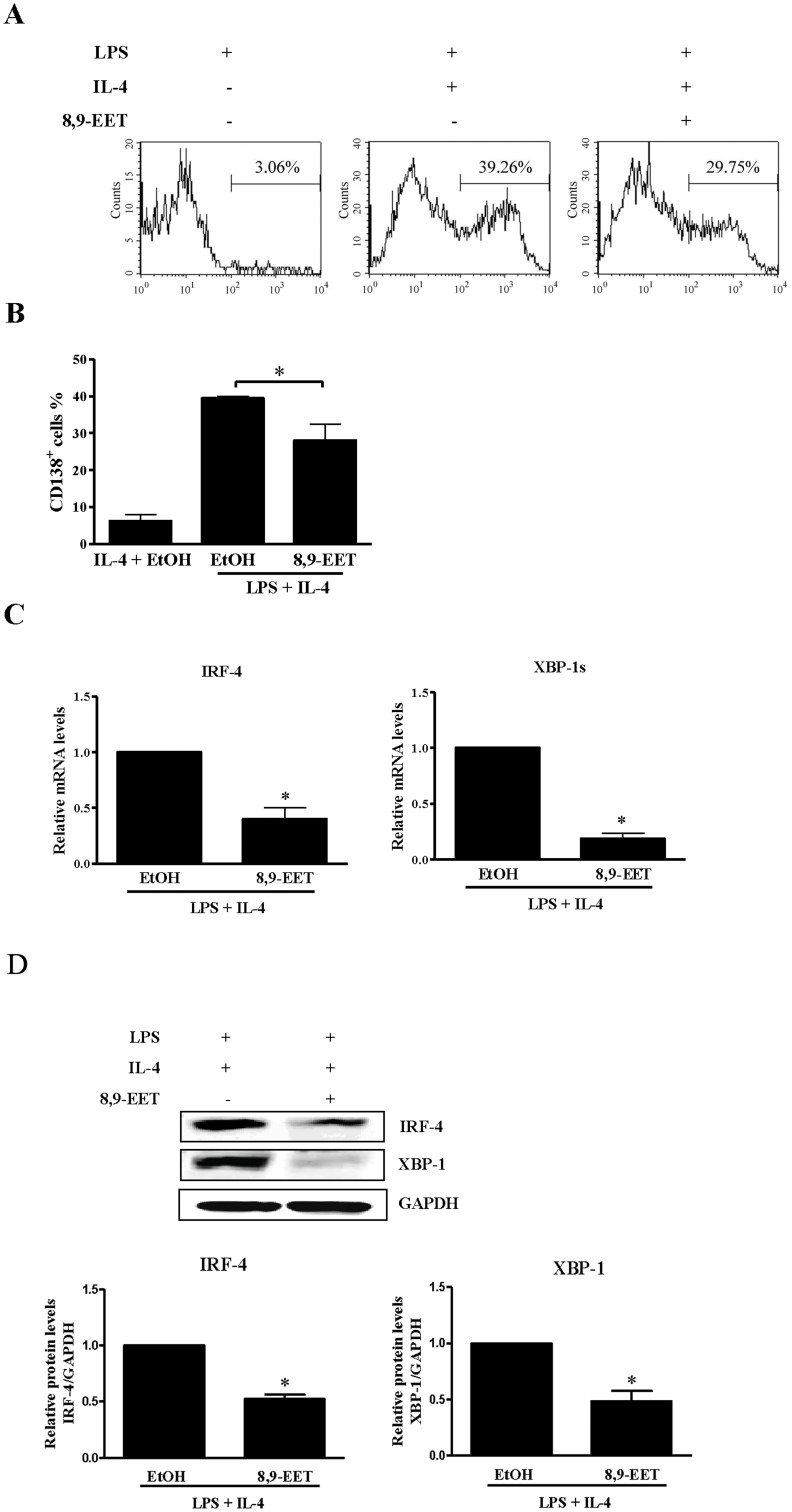
8,9-EET inhibited plasma-cell differentiation of B cells. A. Splenic B cells were stimulated with 5 µg/ml LPS and 50 ng/ml IL-4 with or without 8,9-EET (1 µM) for 3 days, then CD138 expression was analyzed by flow cytometry. B. Quantification of 3 independent experiments of plasma cell differentiation. Quantification of real time PCR and western blot analysis of mRNA (C) and protein (D) expressions of IRF-4 and XBP-1 in B cells cultured with 8,9-EET, 5 µg/ml LPS and 50 ng/ml IL-4 for 3 days. GAPDH was used as the control. Data are means ± SEM of three independent experiments. *, *P*<0.05 vs. no 8,9-EET.

Plasma cells secrete antibodies at a high rate. To investigate the 8,9-EET effect on B-cell differentiation, we measured the surface expression of syndecan-1/CD138, a marker for plasmacytoid cells, 3 days after 8,9-EET stimulation. 8,9-EET markedly inhibited the generation of CD138^+^ cells with LPS plus IL-4 treatment as compared with the control (28.1% ±4.5% vs. 39.5% ±0.5%) (Figure 3A and 3B). B-cell differentiation depends on the expression of several transcription factors, including IRF-4, which induces XBP-1 splicing and plasma-cell formation. Therefore, we measured the mRNA levels of IRF-4 and XBP-1s, the spliced form, in B cells activated by LPS plus IL-4 by real-time PCR. 8,9-EET significantly reduced the mRNA level of IRF-4 (approximately 60%) and XBP-1(s) (approximately 81%) (Figure 3C). Similarly, 8,9-EET significantly decreased the protein expressions of IRF-4 and XBP-1 (Figure 3D). Therefore, 8,9-EET strongly inhibited plasma-cell differentiation in B cells stimulated with LPS plus IL-4.

### 8,9-EET Antagonized Class-switch Recombination

**Figure 4:**
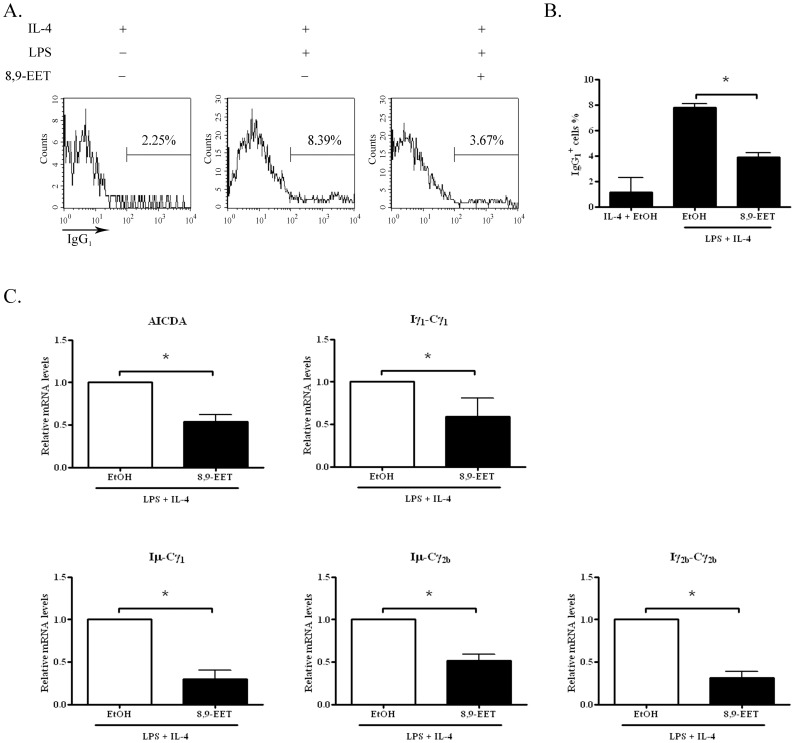
8,9-EET inhibited class switch recombination of B cells. A. Splenic B cells were stimulated with 5 µg/ml LPS and 50 ng/ml IL-4 with or without 8,9-EET (1 µM) for 3 days. Flow cytometry of surface IgG_1_ (sIgG_1_) expression in cells. B. Quantification of 3 independent experiments of sIgG_1_ expression in B cells. C. Real-time RT-PCR analysis of mRNA levels of AICDA, Iγ_1_-Cγ_1_, Iμ-Cγ_1_, Iγ_2b_-Cγ_2b_ and Iμ-Cγ_2b_ in 3-day culture. GAPDH was used as the control. Data are means ± SEM of three independent experiments. *, *P*<0.05 vs. no 8,9-EET.

Upon encountering cognate antigens, B cells are activated to undergo 2 genetic alterations of their immunoglobulin genes, namely, somatic hypermutation and class-switch recombination, which allows for affinity maturation and the generation of different antibody classes (e.g., IgG, IgA and IgE) [26], [27]. To determine whether 8,9-EET affects class-switch recombination, we first examined the expression of surface IgG_1_ (sIgG_1_) in B cells stimulated with LPS plus IL-4 for 3 days. The addition of 8,9-EET to these cultures decreased sIgG_1_^+^ cell production as compared with the control (3.9% ±0.3% vs. 7.8% ±0.3%) (Figure 4A and 4B). Molecular events involved in class-switch recombination include the expressions of germline transcripts (GLTs) and AICDA, followed by deletional switch recombination and expression of Iμ-CH mature transcripts [7]. Therefore, we measured the mRNA levels of GLTs, AICDA and Iμ-CH mature transcripts by real-time PCR. 8,9-EET significantly inhibited the expression of AICDA, Iγ_1_-Cγ_1_, Iμ-Cγ_1_, Iγ_2b_-Cγ_2b_ and Iμ-Cγ_2b_ in B cells stimulated with LPS plus IL-4 (Figure 4C). Thus, 8,9-EET inhibits the class-switch recombination of B cells.

### 8,9-EET Inhibited NF-κB Activation in B Cells

**Figure 5:**
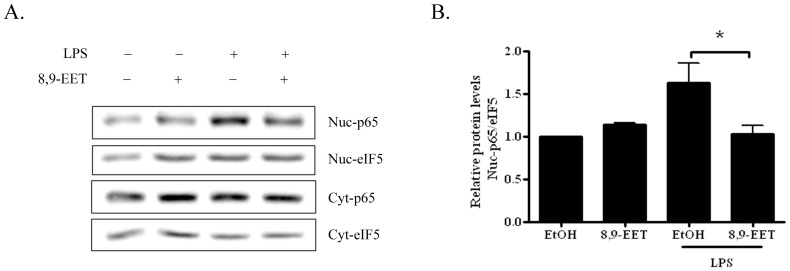
8,9-EET inhibited NF-κB activation in B cells. A. Western blot analysis of cytoplasmic and nuclear protein fraction of B cells measuring NF-κB p65 nuclear translocation after treatment with 1 µM 8,9-EET for 15 min and/or 5 µg/ml LPS for 5 min. Representative results from 3 independent experiments are shown. B. Quantification of 3 independent experiments. Data are means ± SEM. *, *P*<0.05 vs. no 8,9-EET.

To determine the signaling pathway by which 8,9-EET affects B-cell activation and antibody production, we determined whether the activity of NF-κB transcription factor, a major pathway for B-cell activation [28], was involved in 8,9-EET-inhibited B-cell activation. In B-cells pretreated with 8,9-EET and LPS, we determined the LPS-induced nuclear translocation of NF-κB by western blot analysis with anti-p65 NF-κB antibody. Nuclear p65 NF-κB level was increased within 5 min after LPS stimulation and was inhibited by pretreatment with 1 µM 8,9-EET for 10 min (Figure 5A and 5B). A similar result was observed by immunofluorescence analysis (data not shown). Therefore, 8,9-EET might inhibit LPS-induced B-cell activation at least in part by inhibiting the NF-κB signaling pathway.

### Germinal-center Formation was Impaired by 8,9-EET in vivo

**Figure 6:**
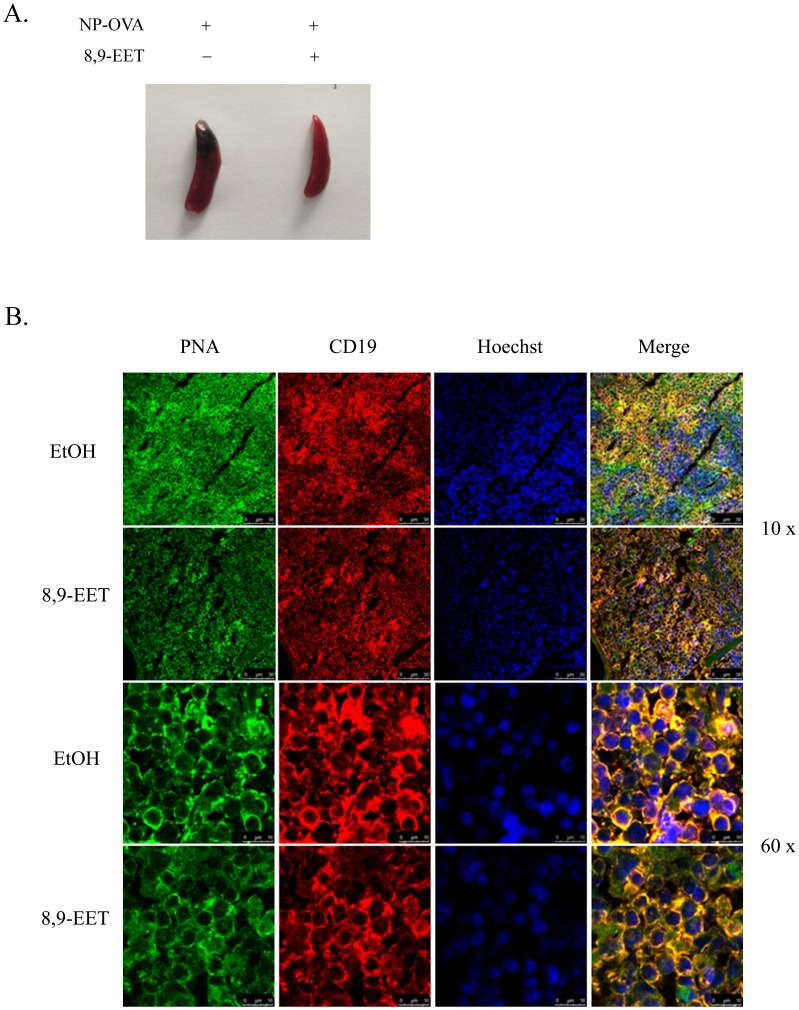
Impaired germinal-center formation in the presence of 8,9-EET *in vivo*. Soluble epoxide hydrolase-deficient (sEH−/−) mice received mini-osmotic pump infusion of 8,9-EET dissolved in a mixture of solvents [PEG 400 (50% vol/vol) + DMSO (35% vol/vol) + ethanol (15% vol/vol)] or vehicle alone. One day later, mice were immunized with 100 µg NP-OVA in alum. A. Sizes of mouse spleens at day 6 postimmunization (8,9-EET infusion: 15 ng/h). B. Confocal microscopy of spleen sections. Splenic germinal centers were stained with CD19 combined with PNA antibody at day 6 postimmunization (8,9-EET infusion: 15 ng/h). n = 4 for each group.

After antigen stimulation, naïve B cells enter into follicles and form germinal centers where B cells rapidly proliferate; germinal centers are the main sites for generation of high-affinity antibody-secreting plasma cells and memory B cells [1]. To determine whether 8,9-EET regulates B-cell function *in vivo*, sEH−/− mice, with reduced EET degradation and treated with or without 8,9-EET were intraperitoneally injected with a T-cell-dependent antigen, 4-hydroxy-3-nitrophenyl acetyl-OVA (NP-OVA). The spleen size of mice with 8,9-EET infusion (15 ng/h) was smaller than that without 8,9-EET at day 6 pos-timmunization (Figure 6A). The colocalization of CD19 and PNA from immunofluorescence analysis of spleens revealed fewer germinal centers and smaller structures with than without 8,9-EET (Figure 6B). Therefore, 8,9-EET inhibited germinal center formation *in vivo*.

## Discussion

EETs are widely considered as endothelium-derived hyperpolarizing factors that modulate a number of biological events in an autocrine or paracrine manner [29], [30] and may play an important role in the regulation of inflammation [15], [31]–[33]. CYP2C9 and sEH are detected in lymphoid tissues [23]. As well, 5,6-EET is principally responsible for hypotonicity-induced responses in B cells [24], [25], so EETs may play a biological role in humoral immunity. We have found that 8,9-EET inhibits B-cell activation induced by LPS with or without IL-4, as demonstrated by decreased IgM and IgG secretion, decelerated cell proliferation, deteriorated cell survival, inhibited plasma-cell formation and restrained class switch recombination. Therefore, B-cell humoral immunity is prevented by 8,9-EET in both normal and ApoE−/− mice. This study indicates a new mechanism for the effect of EETs on immune response, which might be a new therapeutic target for excess B-cell activation.

The effect of 8,9-EET on B cells seems to be specific, because none of the other regioisomers inhibited the B-cell activation. Our findings are consistent with previous reports of the selective effect of different EETs including pre-glomerular endothelium-dependent vasoconstriction (5,6-EET), adenosine-induced vasodilation (11,12-EET) in smooth muscle cells [34], [35], and vasoconstriction (8,9-EET) [36]. Therefore, the position of the epoxide group across the double bonds might be a required molecular conformation for the observed B-cell effect of 8,9-EET.

We further explored the biochemical basis for the inhibition of 8,9-EET on B-cell activation. Many transcription factors such as the cyclic AMP-response element-binding protein [37], NF-κB [15], peroxisome proliferator-activated receptor α (PPARα) [38] and forkhead box O3a [39] are modulated by EETs. NF-κB plays a crucial role in B cell proliferation and antibody production. We found that 8,9-EET inhibited LPS-induced translocation of p65 and enhanced the phosphorylation of IκB (data not shown), which might mediate the effects of 8,9-EET on B-cell function. However, the PPARs pathway might not be involved in the regulation of 8,9-EET in B cells (Figure S1).

Cellular EETs are present in free and phospholipid-bound forms. They are principally metabolized by sEH to corresponding DHETs. The inhibitory effects of 8,9-EET on B cells were demonstrated *in vivo* by direct infusion of 8,9-EET in sEH−/− mice with a C57BL/6 genetic background. After 6 days of 8,9-EET infusion (15 ng/h), sEH−/− mice immunized with NP-OVA in alum showed greatly decreased size of spleens and germinal center structure. Thus, 8,9-EET inhibited the function of B cells both *in vivo* and *in vitro*.

Besides secreting antibodies, activated B cells can also provide co-stimulatory signals for T-cell activation [40], [41]. We found that 8,9-EET decreased the mRNA expression of CD80 and CD86 in B cells (Figure S2), which might lead to weakened and shortened cellular immunity and contribute to the multiple effects of 8,9-EET *in vivo*. However, the mechanism remains to be further investigated.

In conclusion, we have demonstrated that 8,9-EET, a naturally existing epoxide of arachidonic acid, inhibits the function of B cells. Development of more stable analogs of 8,9-EET may provide a new class of specific therapeutic tool for diseases mediated by excess B-cell activation.

## Materials and Methods

### Ethics Statement

This study was carried out in strict accordance with the recommendations in the Guide for the Care and Use of Laboratory Animals of the Health Science Center of Peking University. The protocol was approved by the Committee on the Ethics of Animal Experiments of the Health Science Center of Peking University (Permit Number: 12125). All surgery was performed under sodium pentobarbital anesthesia, and all efforts were made to minimize suffering.

### Reagents and Antibodies

5,6-, 8,9-, 11,12- and 14,15-EET were from Cayman Chemical (Ann Arbor, MI). The anti-mouse p65 antibody was from Santa Cruz Biotechnology (Santa Cruz, CA). The anti-mouse IRF-4 (P173) antibody and anti-mouse XBP-1 antibody were from Cell Signal Technology (Danvers, MA) and Abcam (Cambridge, MA) respectively. Recombinant murine soluble CD40 ligand and recombinant murine interleukin 4 (IL-4) were from Peprotech (Rocky Hill, USA). Anti-mouse IgM F(ab)_2_ was from Jackson Immuno Research Laboratories (West Grove, PA). Lipopolysaccharied (LPS; *Escherichia coli* 055:B5) was from Sigma Co. (St. Louis, MO).

### Mice

We used 8-week-old pathogen-free male C57BL/6 mice and ApoE−/− mice provided by the Animal Center of Peking University Health Science Center (Beijing). Soluble epoxide hydrolase-deficient (sEH−/−) mice were provided by Professor Frank J. Gonzalez (Lab. of Metabolism, NIH, Bldg. 37, Rm. 3E24, Bethesda, MD 20892). All mice were in a C57BL/6 background.

### B-cell Isolation

B cells from mouse spleens were purified by positive selection according to the manufacturer’s protocol (Miltenyi Biotec, Bergisch Gladbach, Germany). Briefly, single-cell suspensions of spleen tissue were centrifuged. After lysis of erythrocytes, single-cell suspensions were incubated with anti-CD19-coated magnetic beads (Miltenyi Biotec, Bergisch Gladbach, Germany) and subjected to a magnetic field to separate B cells. Purified B cells were cultured in RPMI 1640 medium supplemented with 10% fetal bovine serum (FBS), 50 µM β-ME, 10 mM HEPES, 2 mM L-glutamine, 100 U/ml penicillin and 100 µg/ml streptomycin. Cells were kept at 37°C in a 5% CO_2_ incubator for different times.

### Proliferation

Carboxyfluorescein succinimidyl ester (CFSE) staining was used to analyze cell division according to the manufacturer’s protocol (Molecular Probes, Eugene, OR). Briefly, B cells (10^7^/ml) were labeled with 2.5 µM CFSE for 10 min at 37°C in the dark, stopped by 25% FBS, washed 2 times with RPMI 1640 medium containing 10% FBS, then resuspended in RPMI 1640 medium containing 10% FBS. Cells were then plated at 2×10^6^ cells/well in 48-well round-bottom plates. CFSE-labeled cells were cultured under various conditions. Three days later, cells underwent detection by FACS Calibur flow cytometry (BD Biosciences, San Jose, CA).

### Survival Assay

To measure apoptosis, cells stimulated for 2 days under the aforementioned conditions were incubated for 15 min with annexin V (AnnV) and for 5 min with propidium iodide (PI) by use of the Vybrant Apoptosis Assay Kit (Molecular Probes, Eugene, OR) in the dark, and the fraction of subdiploid cells was measured by FACS Calibur flow cytometry.

### B-cell Surface Labeling

Purified mouse B cells (2×10^6^ cells/ml) were cultured in 48-well round-bottom microtiter plates, then treated for 3 days with 5 µg/ml LPS and 50 ng/ml IL-4 in the presence or absence of 8,9-EET and harvested. B cells were incubated with anti-mouse CD138-PE (BD Pharmingen, San Jose, CA) or anti-mouse IgG_1_-PE (Miltenyi Biotec, Bergisch Gladbach, Germany) in cold phosphate buffered saline for 20 min at 20°C, then washed. All samples were assayed by FACS Calibur flow cytometry.

### Antibody Production

Purified mouse B cells (2×10^6^ cells/ml) were cultured in 96-well round-bottom microtiter plates and treated for different days with activating agents in the presence or absence of EETs. Pilot experiments were performed to optimize the doses of EETs and LPS. Supernatants were harvested and the concentrations of IgM and IgG were analyzed by use of mouse-specific ELISA kits (Bethyl Laboratories, West FM, MO).

### Western Blot Analysis

Whole-cell extracts were collected with cell lysis buffer (Beyotime, Jiangsu, China) plus 1 mM PMSF. Cytoplasmic and nuclear protein extracts from B cells were prepared by use of the NE-PER Nuclear and Cytoplasmic Extraction Reagents kit (Pierce, Rockford, IL). Total protein was quantified by bicinchoninic acid protein assay (Pierce, Rockford, IL). Proteins were separated by 10% SDS-PAGE, then electrophoretically transferred onto nitrocellulose membranes, which were incubated with the indicated primary antibody, washed, then incubated with an appropriate IRDyeTM-conjugated second antibody. Specific immunofluorescence bands were detected by use of the Odyssey infrared imaging system (LI-COR Biosciences, Lincoln, NE).

### Quantitative Real-time RT-PCR Analysis

Total RNA from primary mouse spleen B cells was isolated by use of Trizol reagent (Applygen Technologies, Beijing) and reverse transcribed with the reverse transcription system (Promega, Madison, WI). Then the reaction mixture underwent PCR. The nucleotide sequences of primers are shown in Table S1. The amount of PCR products formed in each cycle was evaluated by SYBR Green I fluorescence. Amplification reactions involved the Mx3000 Multiplex Quantitative PCR System (Stratagene, La Jolla, CA). Data were analyzed with use of Stratagene Mx3000 software.

### Immunization of Mice

Healthy sEH−/− mice (6∼10 weeks old) were randomly assigned to 2 groups (n = 6 each) and underwent subcutaneous implantation with a miniosmotic pump for treatment with 8,9-EET dissolved in a mixture of solvents [PEG 400 (50% vol/vol) + DMSO (35% vol/vol) + ethanol (15% vol/vol)] with a flow rate of 15 ng/h for 6 days or vehicle alone. An amount of 100 µg NP-OVA (Biosearch Technologies, Novato, CA) in alum was intraperitoneally injected the next day. Spleens were removed, imaged, embedded in OCT 6 days after immunization, snap-frozen in liquid nitrogen, and stored at −80°C. After acetone fixation, cryosections (5∼6 µm) were blocked with 10% bovine serum albumin in phosphate buffered saline and then washed and incubated with anti-CD19 antibody (Cell Signal Technology, Danvers, MA) over night, then stained with TRITC-conjugated goat anti-rabbit IgG (Rockland Inc., Gilbertsville, PA) and fluoroscein-conjugated peanut agglutinin (FITC-PNA) (Vector Laboratories, Burlingame, CA). Nuclei were counter stained with Hoechst 33342 (Sigma Chemical Co., St. Louis, MO). The stained sections were analyzed by confocal microscopy (DM IRB, Leica Microsystems, Deerfield, IL).

### Statistical Analysis

All data were expressed as mean ± SEM. Data analysis involved unpaired Student *t* test and one-way and two-way ANOVA with GraphPad Prism software (GraphPad, La Jolla, CA) followed by Student-Newman-Keuls tests. *P*<0.05 was considered statistically significant.

## Supporting Information

Figure S1 **PPARs were not involved in the inhibition of 8,9-EET on antibody production of B cells.** ELISA of levels of IgM and IgG (A and B) in the supernatant of cultured B cells from C57BL/6 mice after incubation for 3 days with 1 µM 8,9-EET plus 5 µg/ml LPS and/or 50 ng/ml IL-4 with or without Rosiglitazone, GW9662 or GW0742. ELISA of levels of IgM and IgG (C and D) in the supernatant of cultured B cells from PPARγ+/− and PPARγ+/+ mice after incubation for 3 days with 1 µM 8,9-EET and/or 5 µg/ml LPS with or without IL-4 (50 ng/ml). Data are means ± SEM from 3 independent experiments. *, *P*<0.05 vs. no 8,9-EET.
(TIF)

Figure S2 **8,9-EET inhibited CD80 and CD86 gene expression of B cells.** Splenic B cells were stimulated with 5 µg/ml LPS plus 50 ng/ml IL-4 with or without 8,9-EET (1 µM) for 3 days. Real-time RT-PCR analysis of mRNA expression of CD80 (A) and CD86 (B). GAPDH was used as the control. Data are means ± SEM from 3 independent experiments. *, *P*<0.05 vs. no 8,9-EET.
(TIF)

Table S1 **The nucleotide sequences of primers.** Total RNA from primary mouse spleen B cells was isolated and reverse transcribed with the reverse transcription system. Then the reaction mixture underwent PCR. The nucleotide sequences of primers are shown in this Table S1.
(TIF)
