# Supplementary figures and images for: 8,9-Epoxyeicosatrienoic Acid Inhibits Antibody Production of B Lymphocytes in Mice
Source: PLoS One. 2012 Jul 3;7(7):e40258. doi: 10.1371/journal.pone.0040258 (PMC3389024; doi:10.1371/journal.pone.0040258)

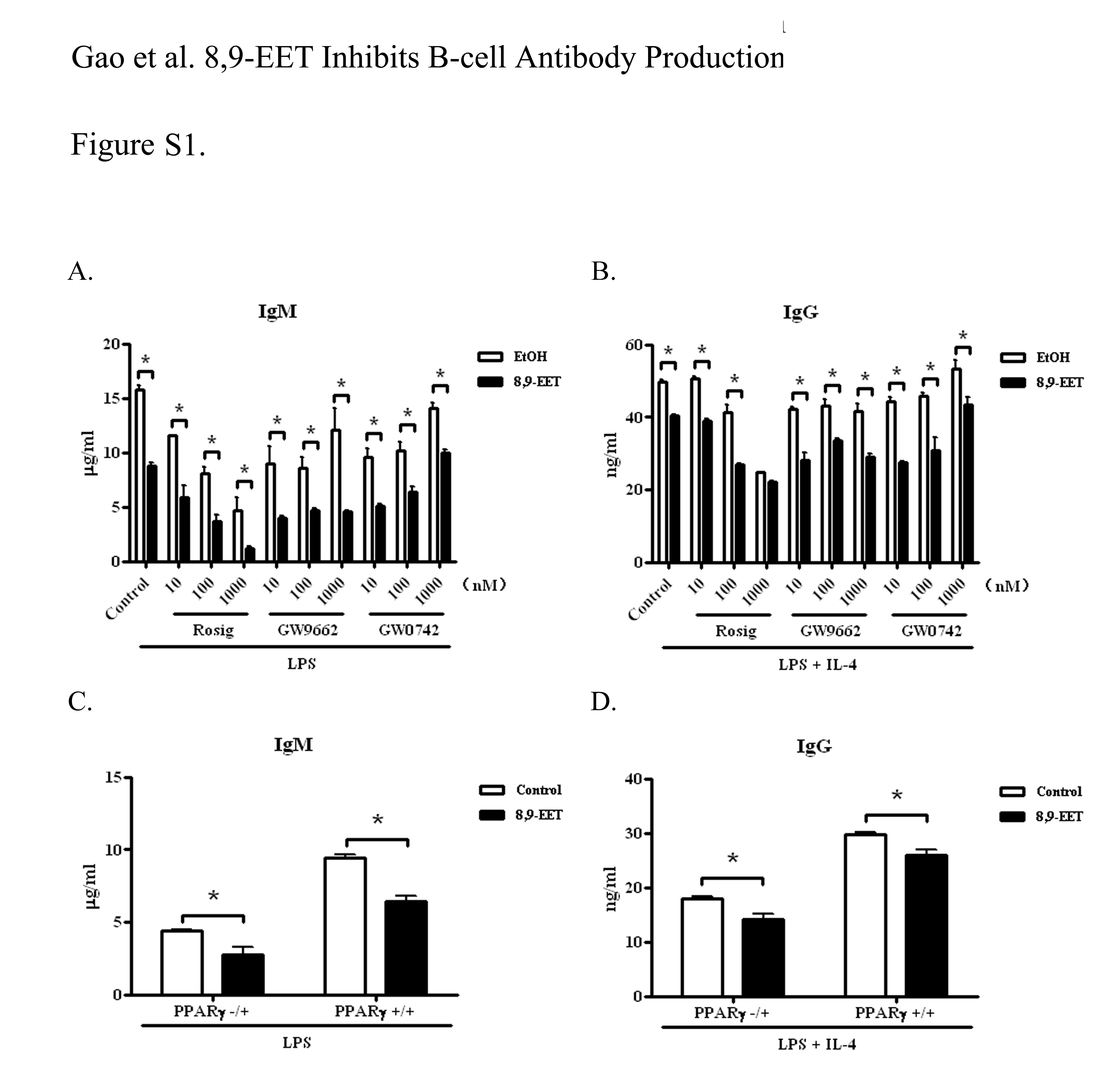

Supplement: Figure S1 — PPARs were not involved in the inhibition of 8,9-EET on antibody production of B cells. ELISA of levels of IgM and IgG (A and B) in the supernatant of cultured B cells from C57BL/6 mice after incubation for 3 days with 1 µM 8,9-EET plus 5 µg/ml LPS and/or 50 ng/ml IL-4 with or without Rosiglitazone, GW9662 or GW0742. ELISA of levels of IgM and IgG (C and D) in the supernatant of cultured B cells from PPARγ+/− and PPARγ+/+ mice after incubation for 3 days with 1 µM 8,9-EET and/or 5 µg/ml LPS with or without IL-4 (50 ng/ml). Data are means ± SEM from 3 independent experiments. *, P<0.05 vs. no 8,9-EET. (TIF) [file pone.0040258.s001.tif]

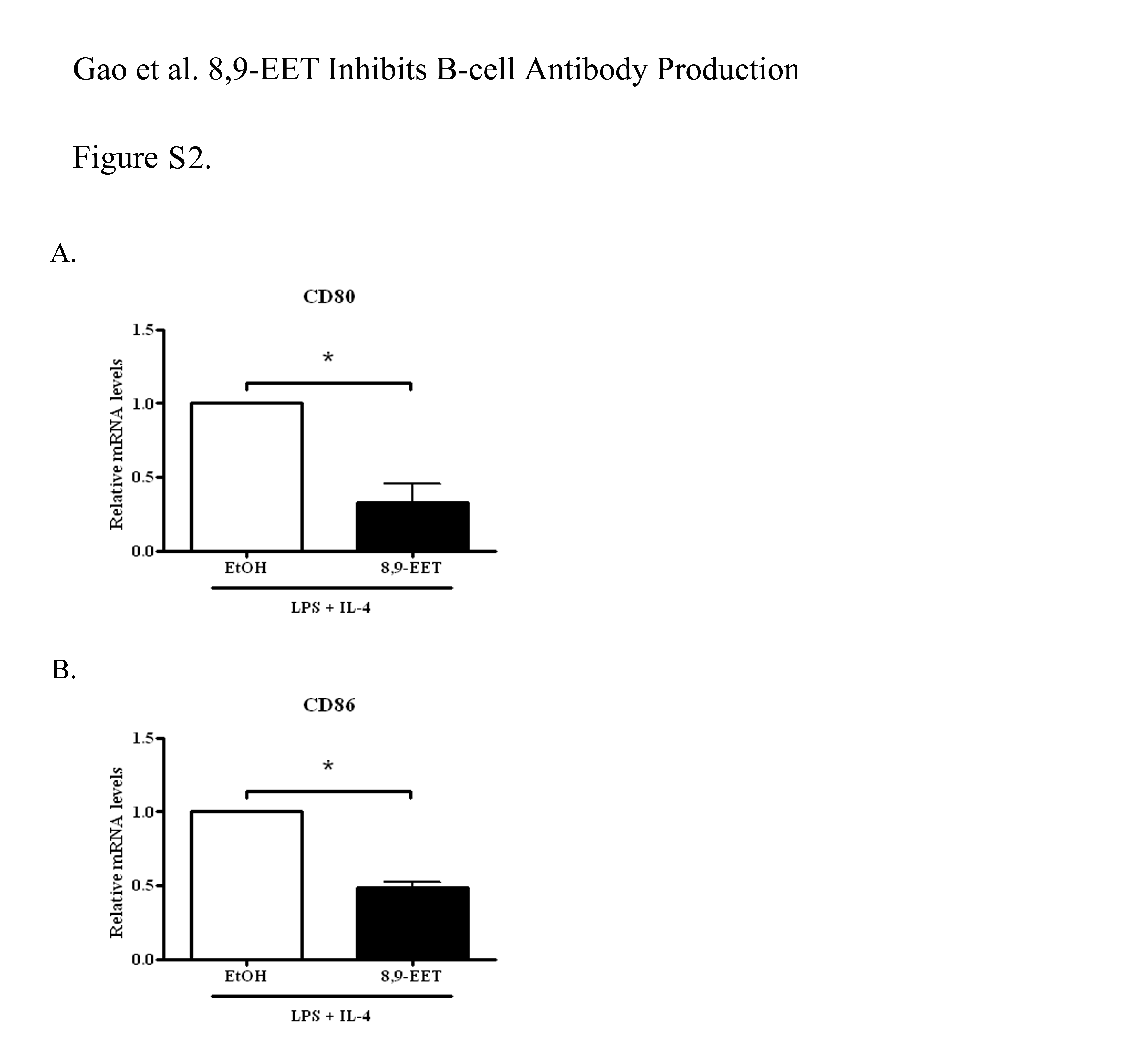

Supplement: Figure S2 — 8,9-EET inhibited CD80 and CD86 gene expression of B cells. Splenic B cells were stimulated with 5 µg/ml LPS plus 50 ng/ml IL-4 with or without 8,9-EET (1 µM) for 3 days. Real-time RT-PCR analysis of mRNA expression of CD80 (A) and CD86 (B). GAPDH was used as the control. Data are means ± SEM from 3 independent experiments. *, P<0.05 vs. no 8,9-EET. (TIF) [file pone.0040258.s002.tif]
